# Galectin-9 Induced Myeloid Suppressor Cells Expand Regulatory T Cells in an IL-10-Dependent Manner in CVB3-Induced Acute Myocarditis

**DOI:** 10.3390/ijms15033356

**Published:** 2014-02-25

**Authors:** Yingying Zhang, Li Jiang, Mengying Zhang, Kun Lv

**Affiliations:** 1 Laboratory of Medicine of The First Affiliated Hospital, Wannan Medical College, 2 Western Zheshan Road, Wuhu 241001, China; E-Mail: ying-micky@163.com; 2 Department of Pharmacy, Institute of Dermatology, Chinese Academy of Medical Sciences, Nanjing 210001, China; E-Mail: jiangli718@aliyun.com; 3 Central Laboratory of The First Affiliated Hospital, Wannan Medical College, 2 Western Zheshan Road, Wuhu 241001, China; E-Mail: zhangmy1983@163.com

**Keywords:** coxsackievirus, myocarditis, myeloid suppressor cells, regulatory T cells

## Abstract

The objective of the study was to explore the effects of galectin-9 on myeloid suppressor cells in Coxsackievirus B3 (CVB3)-induced myocarditis and the possible mechanisms involved. For this purpose, BALB/c male mice were infected with CVB3 on day 0 and then received intraperitoneal (IP) administration of recombinant galectin-9 or phosphate-buffered saline (PBS) daily from day 3 to day 7. The phenotypes and functions of myeloid suppressor cells were evaluated. The role and mechanism of myeloid suppressor cells and subsets in CVB3-induced myocarditis *in vitro* were explored. We found that galectin-9 remarkably increased the frequencies of CD11b^+^Gr-1^+^ cells in the cardiac tissue and spleen with myocarditis. Ly-6G^+^ cells were decreased and Ly-6C^+^ cells were increased in galectin-9-treated mice. In addition, CD11b^+^Gr-1^+^ cells were highly effective in suppressing CD4^+^ T cells. Moreover, our data demonstrate that CD11b^+^Gr-1^+^ cells are capable of expanding regulatory T cells (Tregs) from a preexisting population of natural Tregs, which depends on IL-10 but not TGF-β. Our results indicate that galectin-9 therapy may represent a useful approach to ameliorate CVB3-induced myocarditis.

## 1. Introduction

Viral myocarditis represents a leading cause of sudden death in young adults. Up to 20% of patients with histological evidence of myocarditis will ultimately develop dilated cardiomyopathy, a fatal disease leaving heart transplantation as the only treatment [1–3]. Enteroviruses of the picornavirus family are considered to be the dominant etiology of viral myocarditis, with Coxsackievirus B3 (CVB3) being most common. The murine model of CVB3-induced myocarditis shares many characteristics with human disease. Despite decades of extensive effort, the pathogenesis of viral myocarditis is still not fully understood. Studies in the murine CVB3 myocarditis model have found that although CVB3 can directly destroy myocardium, the strong host Th1 immune responses may play a more critical pathogenetic role in the course of viral myocarditis, verified by the improvement of heart injury and function by immune modulating and inhibiting agents [4–6]. During CVB3 infection, massive myocardial immune cell inflammation could be observed in the mouse model [7]. Such studies have revealed a critical role for CD4^+^ T cells of the Th1 subset as mediators of myocardial inflammation. Opavsky *et al.* [8] found that the severity of disease is attenuated in CD4 knockout mice, confirming the role of CD4^+^ T cells in CVB3-induced myocarditis. Huber *et al.*, found CD4^+^ Th1 cell responses are essential to CVB3-induced myocarditis susceptibility [9,10]. It is worth mentioning that many researchers have found a protective role for interferon γ (IFN-γ) of Th1 cytokines against viral infections by reducing viral replication directly [11–14]. Thus, the role of IFN-γ is still to be elucidated.

In addition, regulatory T cells (Tregs) play a major role in protection against inflammation in the heart, and their alteration by viral infection may contribute substantially to the outcome of myocarditis. Recent studies have shown that autoimmune myocarditis and multi-organ inflammation are controlled by Foxp3^+^ T cells highly expressing the glucocorticoid-induced tumor necrosis factor (TNF) receptor family related protein (GITR) [15,16]. Depletion of the GITR^+^ Tregs allowed activation of autoimmune heart disease. Huber and colleagues described that myocarditis could be overcome by a coxsackievirus variant which maintained and induced Tregs function [5].

Furthermore, inflammatory cytokines play a key role in CVB3-induced myocarditis. IL-1β and IL-18 serve a function in the pathogenesis of CVB3-induced myocarditis in susceptible mice [17]. IL-1 or TNF-α can promote myocarditis in resistant B10.A mice [18]. A previous study has indicated that exogenous administration of IL-4 is found to improve myocardial inflammation and the severity of myocarditis in CVB3 infected mice [4]. As a consequence, either preventing the access of Th1 cells to the heart or blunting their activity represents a potentially valuable form of therapy. Recent studies on some autoimmune lesions caused by pathogenic T cells have indicated that one means of terminating the activity of such T cells is to engage receptors expressed by activated cells that deliver an inhibitory or lethal signal to the cell [19–23]. This effect was also achieved with galectin-9 treatment in some immune-inflammatory diseases such as experimental autoimmune encephalomyelitis and graft-*versus*-host disease.

Galectin-9, one of the β-galactoside binding animal lectins belonging to the galectin family, induces apoptosis of eosinophils, cancer cells, and T cells [24–27]. Galectin-9 preferentially induces apoptosis of activated CD4^+^ T cells through a Ca^+^ influx-calpain-caspase1 pathway [27]. Zhu *et al.*, have recently demonstrated that galectin-9 is a ligand of T cell immunoglobulin- and mucin domain-containing molecule 3 (Tim-3) that was expressed selectively on terminally differentiated Th1 cells, and that galectin-9 induces apoptosis of Tim-3-expressing cells *in vitro* and *in vivo* [20,28]. In fact, exogenous administration of galectin-9 ameliorates experimental allergic encephalitis, an autoimmune disease of the central nervous system [20]. Furthermore, galectin-9 exhibits an anti-inflammatory role in lipopolysaccharide (LPS)-induced inflammation [29] and experimental allergic conjunctivitis (EAC) in mice [30]. More recently it has been shown that galectin-9 ameliorates a mouse collagen-induced arthritis (CIA) model and herpes simplex virus (HSV) induced lesions by regulating the T cell response [31,32]. Our previous study indicated that galectin-9 administration effectively ameliorates CVB3-induced myocarditis by promoting the proliferation of T regulatory cells and the activation of Th2 cells [33].

The present studies were designed to investigate whether Tim-3/galectin-9 plays a role in murine acute myocarditis induced by CVB3 by manipulating the Tim-3/galectin-9 system in one or more cell types involved in causing myocarditis.

## 2. Results and Discussion

### 2.1. Results

#### 2.1.1. Remission of CVB3-Induced Myocarditis by Galectin-9 Administration

**Figure 1.**
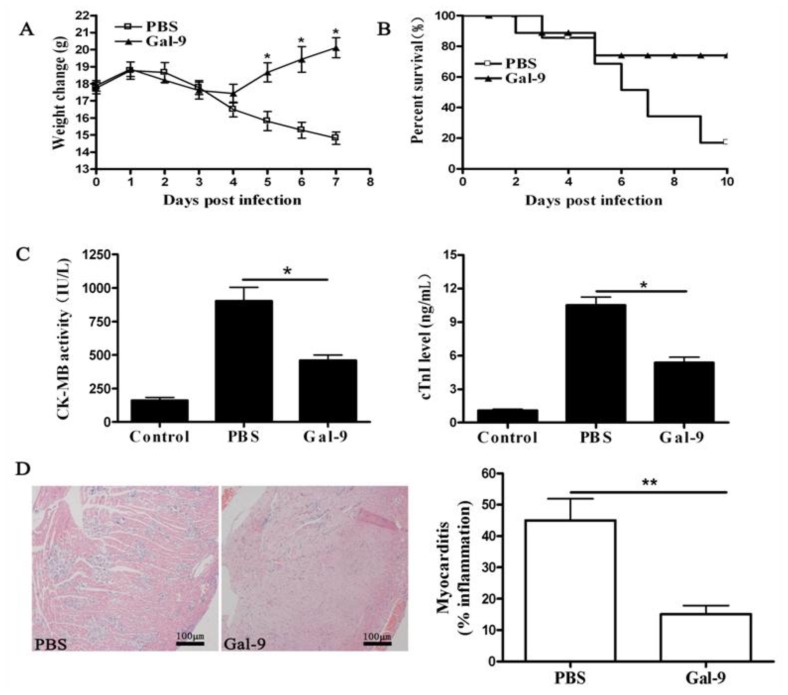

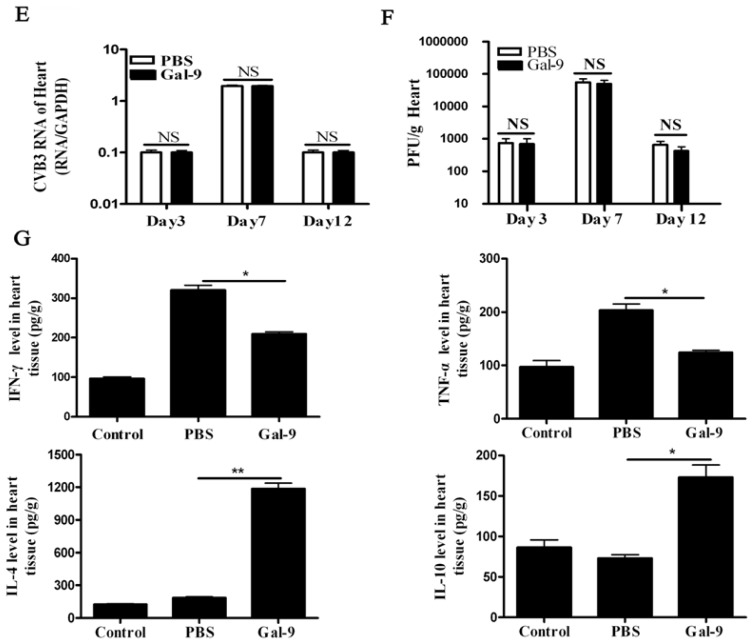
Galectin-9 treatment remarkably alleviated the CVB3 induced cardiac inflammation. BALB/c male mice were infected with CVB3 on day 0 and then received recombinant galectin-9 (*n* = 8) or PBS (*n* = 8) IP daily from day 3 to day 7. The parameters of the viral myocarditis were evaluated including loss of body weight from day 0 to day 7 post-infection (**A**) Activity CK-MB and levels of cTnI (**C**), on day 7 post-infection. The survival rate of mice (*n* = 8) was observed until day 10 post-infection; (**B**) Paraffin sections of heart tissues were prepared on day 7 and cardiac inflammation was revealed by hematoxylin-eosin staining (H&E) staining, magnification: ×100; (**D**) Each group was compared for the number of infiltrated lymphocytes. Meanwhile, the viral tilter in heart of mice was determined by real-time polymerase chain reaction (RT-PCR) (**E**) or plaque assay (**F**); and (**G**) Meanwhile, the heart tissues were homogenized, the expression levels of Th1 and Th2 cytokines were determined by enzyme-linked immunosorbent assay (ELISA assays). Similar results were obtained in three separate experiments. Data show the mean ± SEM. *, *p* < 0.05; **, *p* < 0.01; NS: no statistical significance; Control: normal mice; PBS: PBS treatment in infected mice; and Gal-9: galectin-9 administration in infected mice.

We first investigated whether galectin-9 administration protects mice from CVB3-induced myocarditis. Parameters of myocarditis, including body weight loss, serum creatine kinase MB isoenzyme (CK-MB) activity, serum Troponin I (cTnI) level, pathological features of heart sections, and survival rate as well as cytokines, were carefully studied. It was found that galectin-9 treatment remarkably alleviated the severity of myocarditis. First, mice receiving galectin-9 transiently lost part of their body weight till day 4 post infection and then regained their weight quickly, whereas non-treatment led to a significant weight loss till day 7 (Figure 1A). Consistently, significant decrease of CK-MB activities and low cTnI levels were detected in mice given galectin-9 compared to those of mice receiving PBS (Figure 1C). Finally, histological analysis of heart sections revealed that CVB3 infected mice developed severe myocarditis on day 7 with diffuse inflammation, whereas galectin-9 administration led to a relief of myocardial inflammation showing few restricted mononuclear inflammation foci, indicating a significant therapeutic effect of galectin-9 (Figure 1D). Furthermore, galectin-9 treatment significantly improved the survival rate from about 20% to 80% after CVB3 infection (Figure 1B). These data indicate that *in vivo* galectin-9 administration could effectively rescue mice from lethal myocarditis caused by CVB3 infection. The viral load in heart tissues was also assessed by real-time polymerase chain reaction (PCR) and plaque assay, and it was found that galectin-9 treatment does not significantly change myocardial viral burden (Figure 1E,F), suggesting that the alleviation of viral myocarditis by galectin-9 is not due to the direct down-regulation of viral replication. In addition, the levels of cardiac Th1 cytokines (IFN-γ, TNF-α) were extensively and dramatically decreased in galectin-9 treated mice compared with PBS-treated groups, while Th2 cytokine expression (IL-4, IL-10) was increased significantly in galectin-9 treated mice (Figure 1G), indicating that galectin-9 treatment efficiently impaired Th1 immune responses by significantly reducing Th1 cytokine production, which may ameliorate the CVB3-induced myocardial injury.

#### 2.1.2. The Systemic and Local Immune Responses after Galectin-9 Treatment

**Figure 2.**
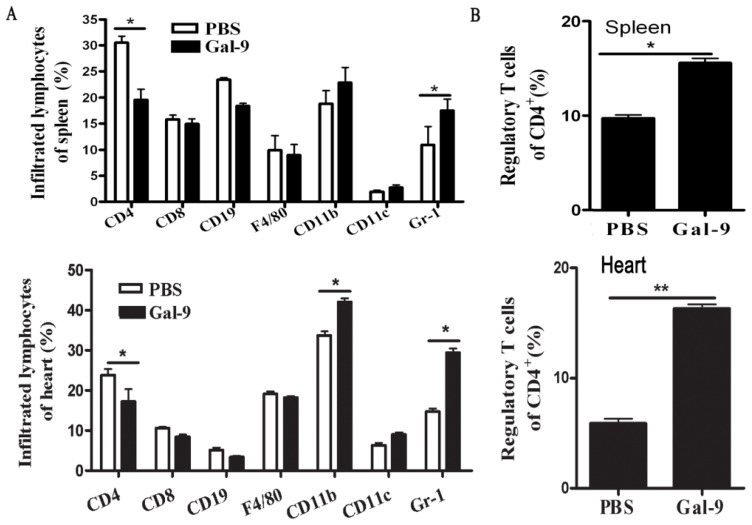
Influence of galectin-9 on cellular immune response during CVB3 infection. BALB/c male mice were infected with CVB3 on day 0 and received recombinant galectin-9 (100 μg/mL, *n* = 8) or PBS (*n* = 8) IP daily from day 3 to day 7. Then the splenocytes and the heart-infiltrated cells were isolated on day 7 after enzymatic digestion and analyzed for CD4, CD8, CD11b, CD11c, CD19, F4/80 and regulatory T cells by FACS. A statistically significant difference in these groups is indicated (**A** and **B**). Similar results were obtained in three separate experiments. Data show the mean ± SEM. *, *p* < 0.05; **, *p* < 0.01.

We first performed experiments to clarify whether galectin-9 modulates the balance of T immune response and influences the number of Tregs. Figure 2 shows that galectin-9 administration significantly decreased the percentage of CD4^+^ T cells, whereas it remarkably increased the percentage of Gr-1^+^ cells as well as Tregs in the spleen and the heart during CVB3-induced myocarditis (Figure 2A,B).

#### 2.1.3. Frequency and Phenotypes of CD11b^+^Gr-1^+^ Myeloid Suppressor Cells in Galectin-9-Treated Mice

**Figure 3.**
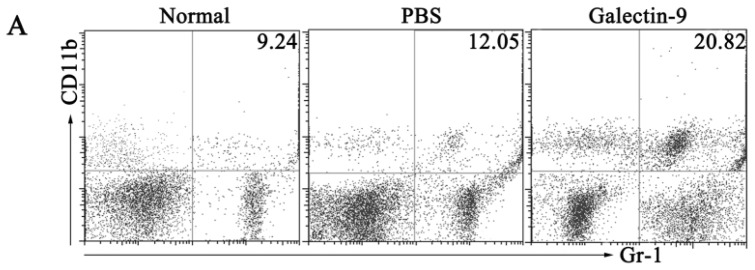

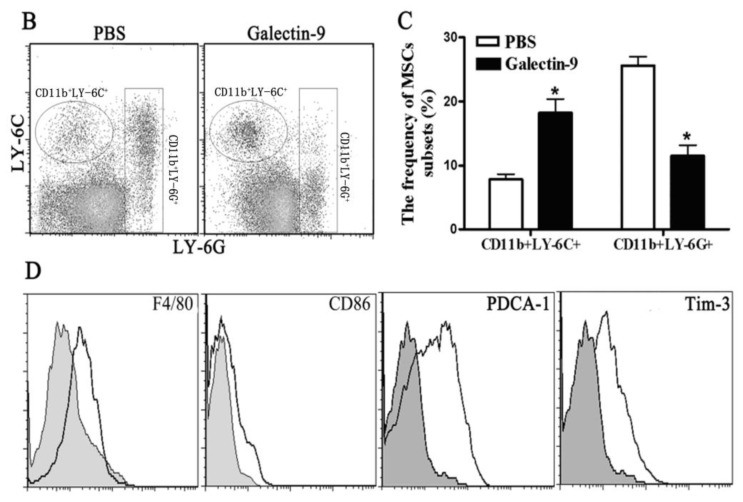
Effects of galectin-9 administration on CD11b^+^Gr-1^+^ myeloid suppressor cells. BALB/c male mice were infected with CVB3 on day 0 and received recombinant galectin-9 (100 μg/mL, *n* = 8) or PBS (*n* = 8) IP daily from day 3 to day 7. (**A**) Then the splenocytes were isolated on day 7 after enzymatic digestion and analyzed for CD11b^+^Gr-1^+^ myeloid suppressor cells by FACS; and (**B**–**D**) Phenotypic characterization of CD11b^+^Gr-1^+^ myeloid suppressor cells from galectin-9-treated animals with respect to the expression of Ly6G, Ly6C, CD86, F4/80, PDCA-1 and Tim-3 is shown; Data show the mean ± SEM. *, *p* < 0.05. Similar results were obtained in three separate experiments and the representative results are indicated in (**A**), (**B**) and (**D**).

To determine which types of Gr-1^+^ cells were increased in galectin-9-treated mice, we first compared CD11b and Gr-1 expression in splenocytes between PBS-treated and galectin-9-treated mice. In FACS analysis, the frequency of CD11b^+^Gr-1^+^ cells in the spleen of galectin-9-treated mice was significantly higher than in PBS-treated mice 7 days after CVB3 infection (Figure 3A). Moreover, Ly-6G^+^ cells were decreased and Ly-6C^+^ cells were increased in galectin-9-treated mice (Figure 3B,C), indicating that galectin-9 induces an increase in CD11b^+^Ly-6G^−^Ly-6C^+^ cells (probably monocyte/macrophage lineage cells). In addition, CD11b^+^Ly-6C^+^ cells were further found to co-express F4/80, CD86, and PDCA-1 (Figure 3D). Because galectin-9 is a ligand of Tim-3, we studied Tim-3 expression on CD11b^+^Ly-6C^+^ cells from galectin-9-treated mice. FACS analysis revealed that CD11b^+^Ly-6C^+^ cells expressed Tim-3 (Figure 3D). However, whether Tim-3/galectin-9 interaction is involved in the expansion of CD11b^+^Ly-6C^+^ cells, remains to be established.

#### 2.1.4. Functional Analyses of CD11b^+^Gr-1^+^ Myeloid Suppressor Cells in Galectin-9-Treated Mice

**Figure 4.**
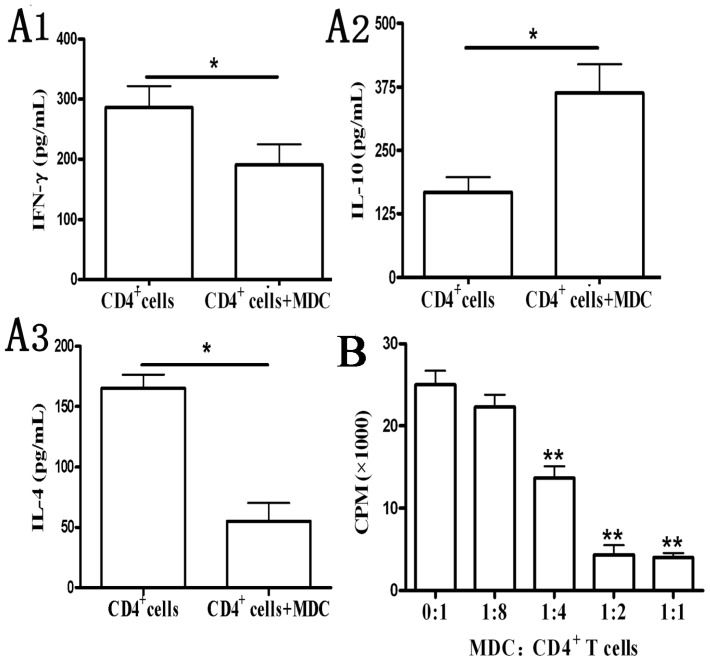
Functional analyses of CD11b^+^Gr-1^+^ myeloid suppressor cells in galectin-9-treated mice. Splenic CD4^+^ T cells from normal BALB/c mice were pre-activated with plate-bound anti-CD3/anti-CD28 (both 2 μg/mL) for 24 h. Splenic CD11b^+^Gr-1^+^ myeloid suppressor cells were purified on day 7 from Gal-9-treated mice. Pre-activated CD4^+^ T cells, in the presence of anti-CD3/anti-CD28 stimulation, were either cultured alone or co-cultured at increasing ratios from 1:8 to 1:1 with CD11b^+^Gr-1^+^ cells. Both CD4^+^ and CD11b^+^Gr-1^+^ cells were added at 2 × 10^5^/well. Supernatant samples from CD4^+^ cell culture and CD4^+^ cell/CD11b^+^Gr-1^+^ cells co-culture (1:1) were collected 24 h after adding myeloid suppressor cells, and the concentrations of various cytokines were examined. (**A1**–**A3**) Proliferation assay was performed after 24 h; and (**B**) Similar results were obtained in three separate experiments. Data show the mean ± SEM. *, *p* < 0.05; **, *p* < 0.01, compared with CD4^+^ cells alone. MDC: myeloid suppressor cells.

Next, the function of the CD11b^+^Gr-1^+^ cells from galectin-9-treated mice was analyzed. We found that upon coculturing the 2 populations, there is significantly increased IL-10 secretion as well as decreased IFN-γ and IL-4 production (Figure 4A). In addition, CD11b^+^Gr-1^+^ cells were highly effective in suppressing CD4^+^ T cells, because a 1:2 coculture could still suppress ~90% of the proliferation, and 1:4 ratio culture suppressed about half of the T cell proliferation (Figure 4B).

**Figure 5.**
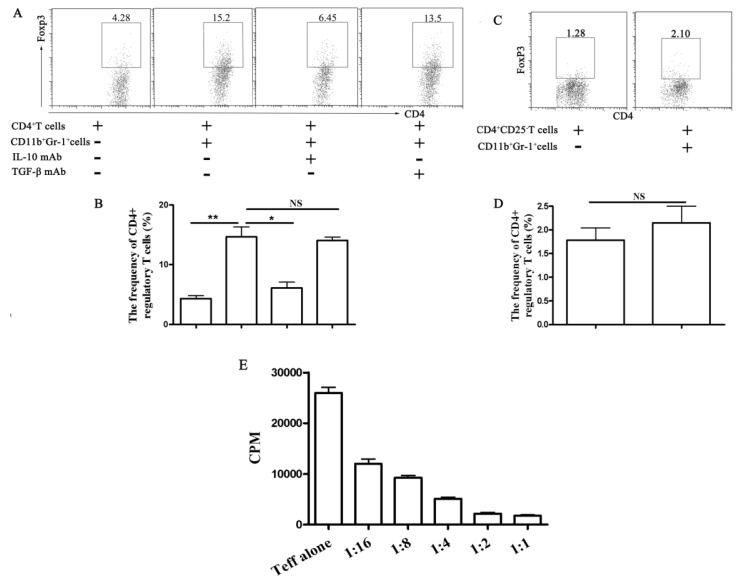
The effect of CD11b^+^Gr-1^+^ myeloid suppressor cells in galectin-9-treated mice on the induction of Tregs. For induction of Tregs *in vitro*, CD4^+^ or CD4^+^CD25^−^ T cells were isolated from splenocytes using the CD4^+^CD25^+^ T cell Isolation Kit. The isolated CD4^+^ T cells in RPMI 1640 with 10% heat-inactivated fetal bovine serum, IL-2 (20 ng/mL), anti-CD28 (2 μg/mL) were distributed into anti-CD3 coated 96-well plate at 2 × 10^5^ cells/well in the presence or absence of TGF-β mAb (1 μg/mL), IL-10 mAb (20 ng/mL) and co-cultured with isolated CD11b^+^Gr-1^+^ myeloid suppressor cells for 4 days at 37 °C in an atmosphere containing 5% CO_2_. Then the percentage of CD4^+^CD25^+^Foxp3^+^ cells was detected by FACS (**A**–**D**), and (**E**) The ability to suppress the proliferation of CD25^−^CD4^+^ T cells was tested with FACS-sorted populations of expanded CD4^+^CD25^high^ T cells. CD4^+^CD25^−^ T cells were incubated in the presence of anti-CD3 and anti-CD28. Proliferation was determined with ^3^H-thymidine incorporation. In co-incubation, increasing numbers of CD4^+^CD25^high^ T cells were added to a constant number of CD4^+^CD25^−^ T cells at the ratios indicated. Data show the mean ± SEM. *, *p* < 0.05; **, *p* < 0.01; NS: no significance; and Teff: effector T cells. Similar results were obtained in three separate experiments and the representative results are indicated in (**A**) and (**C**).

Because we saw an increase in IL-10 production by T cells upon co-incubation with CD11b^+^Gr-1^+^ cells, we further examined the T cells from the co-cultures for regulatory phenotypes. As shown in Figure 5A,B, CD11b^+^Gr-1^+^ cells promoted the expansion of Tregs. The effect depended on IL-10 but not TGF-β, because the presence of an IL-10-neutralizing antibody resulted in a 90% decrease in expansion of Tregs. The experiments described above show that mesenchymal stem cells (MSCs) can expand the pool of Tregs. However, they do not establish whether this Foxp3^+^ population is derived from the conversion of Foxp3^−^ effector T cells or from the selective expansion of a preexisting population of Foxp3^+^ Tregs. To answer these questions, CD4^+^CD25^−^ naive T cells were purified and admixed with CD11b^+^Gr-1^+^ cells sorted from galectin-9-treated mice, and then the Foxp3^+^ Tregs were assayed. As expected, CD11b^+^Gr-1^+^ cells from galectin-9-treated mice did not increase the Tregs (Figure 5C,D). Moreover, the induced CD4^+^CD25^high^ T cells exhibited a stepwise inhibition of CD4^+^CD25^−^ T effector cells (Figure 5E) indicating the potent inhibitory function of these induced CD4^+^CD25^high^ Tregs.

### 2.2. Discussion

Viral myocarditis is an inflammation of the myocardium that follows enterovirus or adenovirus infections. It is the composite result of both virus infection and host uncontrolled immune reactions [34]. During the early viremia period, CVB3 infects heart tissues by receptor-mediated endocytosis [35] and may cause myocardiocyte dysfunction by disrupting dystrophin-sarcoglycan complex or cleaving eukaryotic initiation factor-4 [36]. At later stage of infection, proinflammatory cytokines and Th1 cells are robustly expressed which result in the massive inflammation and aggravated injury in heart [37]. Therefore, re-establishing an immune balance by modulating CD4^+^ T cells is a potential therapeutic strategy for viral myocarditis.

It is well-established that in the sub-acute stage of CVB3 myocarditis (day 4–14), excessive immune responses become the dominant damage factor instead of virus virulence [38]. Th1-dominant immunity has been considered as one of the important mechanisms in the development of CVB3 myocarditis, and the shift of Th1 to Th2 immune response could alleviate myocarditis severity [39]. In addition, Tregs play a major role in protection against inflammation in the heart, and their alteration by viral infection may contribute substantially to the outcome of myocarditis [5]. Sylvia *et al.*, have indicated that reduced inflammation in the heart of females following CVB3 infection is due to increased Tim-3 expression on APC, resulting in increased CTLA-4 expression and Tregs populations [40].

Since the main T cell subset responsible for orchestrating heart injury appear to be CD4^+^ T cells of Th1 type with perhaps some involvement by Th17 CD4^+^ T cells, a logical approach to therapy would be to suppress or delete the function of activated CD4^+^ T cell subsets and increase the representation of cells that express regulatory function. We show herein that this outcome can be achieved by therapy, systemic or local, with the lectin family member galectin-9. This molecule, which is a natural product of cell types such as several cells of the innate immune system, endothelial cells, and epithelial cells, acts as a ligand to the inhibitory molecule Tim-3. Tim-3 is expressed on the surface of both Th17 cells and Th1 cells which are critically involved in initiation of inflammatory and autoimmune disease [41]. Zhu *et al.*, have revealed that the Tim-3-galectin-9 pathway has evolved to ensure effective termination of effector Th1 cells [20]. Thus, it is possible that galectin-9 plays a role in the pathology of cardiovascular disease.

Galectin-9 is considered a Th1-regulator and plays a critical role in many Th1-mediated diseases, such as experimental allergic encephalitis [20], LPS-induced inflammation [29], experimental allergic conjunctivitis [30], collagen-induced arthritis [31] and HSV-induced lesions [32]. Seki *et al.* [31] has recently proved that galectin-9 significantly up-regulates TGF-β induced Foxp3 expression and promotes differentiation into Tregs *in vitro*. Thus, regulating the Tim-3-galectin-9 signaling pathway may significantly impair the induction and recruitment of Th1 cells to the local tissue site, weaken the secondary tissue injury and improve organ function.

MSCs have been characterized as a population of cells that can negatively regulate T-cell function. MSCs are a heterogeneous population of myeloid cells including macrophages, granulocytes, and other cells that express both Gr-1 and CD11b in mice and suppress immune responses *in vivo* and *in vitro* [42]. In this study, we found that CD11b^+^Gr-1^+^ cells, which are markedly increased in the spleen after CVB3 infection, are highly suppressive for activated CD4^+^ T cells. Moreover, Ly-6G^+^ cells were decreased and Ly-6C^+^ cells were increased in galectin-9-treated mice. These cells express F4/80, CD86 and Tim-3, up-regulate IL-10 production and down-regulate IFN-γ and IL-4 level in CD4^+^ T cells upon co-culturing. It has been shown that immature CD11b^+^Gr-1^+^ myeloid cells in mice induce Tregs *in vitro* [43], but a direct link between MSCs and Tregs has not been demonstrated so far. Our data demonstrate that MSCs are capable of expanding Tregs from a preexisting population of natural Tregs, which depends on IL-10 but not TGF-β. Moreover, the expanded CD4^+^CD25^high^ T cells exhibited a stepwise inhibition of CD4^+^CD25^−^ T effector cells, indicating the potent inhibitory function of these Tregs.

The results of this study highlight the importance in delineating the contribution of inflammation and viral replication to the development of viral myocarditis. Clinicians struggle with determining whether patient treatment should be aimed at reducing viral replication in the heart or reducing inflammation and proinflammatory cytokines.

## 3. Experimental Section

### 3.1. Animals

Six-week-old male BALB/c mice (H-2d MHC haplotype) were purchased from the experimental animal center of the Chinese Academy of Science (Shanghai, China). All animals were housed in pathogen-free mouse colonies and all animal experiments were performed according to the guidelines for the Care and Use of Laboratory Animals (Ministry of Health, Beijing, China, 1998) and the guidelines of the Laboratory Animal Ethical Commission of Wannan Medical College (Anhui, China).

### 3.2. Virus

The original stock of CVB3 (Nancy strain) was maintained by passage through Hela cells (ATCC number: CCL-2). Virus titer was routinely determined prior to infection by a 50% tissue culture infectious dose (TCID_50_) assay of HeLa cell monolayers according to previously published procedures [44].

### 3.3. Myocarditis

Mice were infected by an intraperitoneal (IP) injection of 0.1 mL of phosphate-buffered saline (PBS) containing approximately 1 × 10^3^ plaque forming units (PFU) of the virus on day 0. Recombinant human galectin-9 (100 μg/mL) (Cosmo Bio, Tokyo, Japan) or PBS was injected IP daily from day 3 to day 7 and tissue or cells were collected on day 7. Hearts were cut longitudinally and fixed in 10% phosphate-buffered formalin and embedded in paraffin. Sections 5 μm thick were cut at various depths in the tissue section and stained with H&E to determine the level of inflammation. Sections were examined by two independent investigators in a blinded manner, and myocarditis was assessed as the percentage of the heart section with inflammation compared with the overall size of the heart section, with the aid of a microscope eyepiece grid.

### 3.4. Real-Time Polymerase Chain Reaction (RT-PCR)

Total RNA was extracted from heart tissue by Trizol reagent (Invitrogen, Carlsbad, CA, USA) and reverse transcribed into cDNA. To determine the myocardial viral RNA load, total RNA was reverse transcribed to cDNA using specific primer (5′-CAC CGG ATG GCC AAT CCA-3′) and then subjected to real-time PCR using CVB3 primers (5′-ATC AAG TTG CGT GCT GTG-3′ and 5′-TGC GAA ATG AAA GGA GTG T-3′). The expression of CVB3 mRNA was normalized to glyceraldehyde-3-phosphate dehydrogenase (GAPDH) expression.

### 3.5. Serological Index of Myocarditis

Serum MB isoenzyme of creatine kinase (CK-MB) activities were measured on chemistry analyzer DXC800 (Beckman Coulter, Inc., Indianapolis, IN, USA) and cTnI was measured on immunology analyzer DXI800 (Beckman Coulter, Inc.).

### 3.6. Cytokines Enzyme-Linked Immunosorbent Assay (ELISA)

IFN-γ, TNF-α, IL-4 and IL-10 expression levels in the supernatant of co-cultured cells were determined by enzyme-linked immunosorbent assay (ELISA) (R&D System, Minneapolis, MN, USA) following the manufacturer’s instructions. In brief, diluted capture antibody was added in a volume of 100 μL to each well of the ELISA plate (Costar, Cambridge, MA, USA). Plates were sealed and incubated overnight at 4 °C. Plates were washed (300 μL of PBS-Tween, three times), blocked and emptied. Samples and standards were added to triplicate wells (100 μL/well) and plates were incubated at room temperature (RT) for 2 h. After washing, biotinylated detection antibody was added for 60 min at RT, followed by 100 μL of horseradish peroxidase avidin for 30 min at RT. 3,3′,5,5′-Tetramethylbenzidine (TMB) substrate (Merck, Darmstadt, Germany) was added to each well. After 10 min at RT, 50 μL of stop solution was added and absorbance was measured at a wave-length of 450 nm.

### 3.7. Myeloid Cell Isolation and Culture

Splenocytes were isolated after red blood cell (RBC) lysis. CD11b^+^ cells were positively selected using CD11b MACS beads (Miltenyi Biotec, Bergisch Gladbach, Germany), and then stained with Gr-1 mAb. Gr-1^+^ myeloid cell populations were isolated through cell sorting. Purified myeloid cells were cultured in RPMI 1640 medium containing 10% FBS, with the addition of glutamine, sodium pyruvate, nonessential amino acid, and antibiotics.

### 3.8. FACS Analysis

Individual cell suspensions were pooled from heart and spleen. Cells were stained with the following mAbs (eBioscience Inc., San Diego, CA, USA) diluted in 1% FBS in PBS: CD4, CD8, CD11b, CD11c, CD19, Gr-1, Ly6G, Ly6C, CD86, F4/80, PDCA-1, Tim-3, CD25, and Foxp3. For intracellular staining, cells were fixed and permeabilized using fixation buffer and permeabilization solution or an anti-mouse Foxp3 staining kit (eBioscience Inc.). Cell fluorescence was measured using FACS and data was analyzed using Cell Quest software (BD Biosciences, San Jose, CA, USA).

### 3.9. T Cell Proliferation and Cytokine Assays

Flat bottom 96-well plates were coated with anti-CD3/anti-CD28 (both 2 μg/mL) for 3 h at 37 °C. Splenic CD4^+^ T cells were purified using specific MACS beads (Miltenyi Biotec, Bergisch Gladbach, Germany), and then stimulated with plate-bound anti-CD3/anti-CD28 at 2 × 10^5^ cells/well for 24 h. These activated T cells were either cultured alone or co-cultured with isolated CD11b^+^Gr-1^+^ myeloid suppressor cells, in the presence of plate-bound anti-CD3/anti-CD28 stimulation. After 24 h, 1 μCi [^3^H]thymidine was added into each well, and cells were harvested 16 h later. To measure the cytokine concentration in culture, supernatants were collected before adding [^3^H]thymidine, and assays were conducted by ELISA assay (R&D System) following the manufacturer’s instructions.

### 3.10. Induction of Tregs *in Vitro*

Splenocytes were isolated from eight week-old normal mice. CD4^+^ or CD4^+^CD25^−^ T cells were isolated from splenocytes using a CD4^+^CD25^+^ T cell Isolation Kit (Miltenyi Biotec) according to the manufacturer’s instruction. Purity of CD4^+^ T cells was about 95%. The isolated CD4^+^ T cells in RPMI 1640 (Sigma-Aldrich, St. Louis, MO, USA) with 10% heat-inactivated fetal bovine serum, IL-2 (20 ng/mL, R&D systems, Minneapolis, MN, USA), anti-CD28 (2 μg/mL, Becton Dickinson, San Jose, CA, USA) were distributed into anti-CD3 coated 96-well plate at 2 × 10^5^ cells/well in the presence or absence of TGF-β mAb (1 μg/mL, R&D systems), IL-10 mAb (20 ng/mL, ProSpec Ltd., Ness-Ziona, Israel) and co-cultured with isolated CD11b^+^Gr-1^+^ myeloid suppressor cells for four days at 37 °C in an atmosphere containing 5% CO_2_. Then the percentage of CD4^+^CD25^+^Foxp3^+^ cells was detected by FACS.

### 3.11. Suppression Assays

To measure suppressive activity of expanded CD4^+^CD25^high^ T cells, 5 × 10^4^ CD4^+^CD25^−^ cells sorted by FACS were treated with 2 μg/mL anti-CD3 (Becton Dickinson) and anti-CD28 (Becton Dickinson) as effector cells, with or without Tregs at different ratios (1:1, 2:1, 4:1, 8:1, and 16:1) for 72 h in a complete medium containing RPMI 1640 (Sigma-Aldrich, St. Louis, MO, USA). [^3^H]thymidine (0.5 μCi/well) was added 18 h prior to cell collection. Proliferative responses were measured using [^3^H]thymidine incorporation assay.

### 3.12. Statistical Analysis

Data are shown as the mean ± SEM. Statistical analysis of the data was performed with the two-tailed independent Student’s *t*-test and ANOVA analysis using SPSS, version 12.0 (SPSS Inc., Chicago, IL, USA). *p* < 0.05 was considered statistically significant.

## 4. Conclusions

The present study demonstrates that galectin-9 may play a crucial role in CVB3-induced myocarditis and that it may represent a novel therapeutic candidate that is able to suppress autoimmune inflammation by regulating T cell differentiation and the balance of pathogenic and regulatory T cells, such that production of pro-inflammatory cytokines are inhibited and anti-inflammatory cytokines are enhanced.
